# Immune-Mediated Diseases Associated With Cancer Risks

**DOI:** 10.1001/jamaoncol.2021.5680

**Published:** 2021-12-02

**Authors:** Ming-ming He, Chun-Han Lo, Kai Wang, Georgios Polychronidis, Liang Wang, Rong Zhong, Markus D. Knudsen, Zhe Fang, Mingyang Song

**Affiliations:** 1 Department of Medical Oncology, Sun Yat-sen University Cancer Center, State Key Laboratory of Oncology in South China, Collaborative Innovation Center for Cancer Medicine, Guangzhou, China; 2 Department of Epidemiology, Harvard T.H. Chan School of Public Health, Boston, Massachusetts; 3 Clinical and Translational Epidemiology Unit, Massachusetts General Hospital, Harvard Medical School, Boston; 4 Division of Gastroenterology, Massachusetts General Hospital, Harvard Medical School, Boston; 5 Department of General, Visceral and Transplantation Surgery, University of Heidelberg, Heidelberg, Germany; 6 Study Centre of the German Surgical Society, University of Heidelberg, Heidelberg, Germany; 7 Center of Gastrointestinal Surgery, First Affiliated Hospital, Sun Yat-sen University, Guangzhou, China; 8 Section for Colorectal Cancer Screening, Cancer Registry of Norway, Oslo, Norway; 9 Division of Surgery, Department of Transplantation Medicine, Inflammatory Diseases and Transplantation, Norwegian PSC Research Center, Oslo University Hospital, Oslo, Norway; 10 Department of Nutrition, Harvard T.H. Chan School of Public Health, Boston, Massachusetts

## Abstract

**Importance:**

Immune regulation is important for carcinogenesis; however, the cancer risk profiles associated with immune-mediated diseases need further characterization.

**Objective:**

To assess the prospective association of 48 immune-mediated diseases with the risk of total and individual cancers and the prospective association of organ-specific immune-mediated diseases with the risk of local and extralocal cancers.

**Design, Setting, and Participants:**

This prospective cohort study used data from the UK Biobank cohort study on adults aged 37 to 73 years who were recruited at 22 assessment centers throughout the UK between January 1, 2006, and December 31, 2010, with follow-up through February 28, 2019.

**Exposures:**

Immune-mediated diseases.

**Main Outcomes and Measures:**

The association of immune-mediated diseases with risk of cancer was assessed with multivariable hazard ratios (HRs) and 95% CIs after adjusting for various potential confounders using time-varying Cox proportional hazards regression. Heterogeneity in the associations of organ-specific immune-mediated diseases with local and extralocal cancers was assessed using the contrast test method.

**Results:**

A total of 478 753 participants (mean [SD] age, 56.4 [8.1] years; 54% female) were included in the study. During 4 600 460 person-years of follow-up, a total of 2834 cases of cancer were documented in 61 496 patients with immune-mediated diseases and 26 817 cases of cancer in 417 257 patients without any immune-mediated diseases (multivariable HR, 1.08; 95% CI, 1.04-1.12). Five of the organ-specific immune-mediated diseases were significantly associated with higher risk of local but not extralocal cancers: asthma (HR, 1.34; 95% CI, 1.14-1.56), celiac disease (HR, 6.89; 95% CI, 2.18-21.75), idiopathic thrombocytopenic purpura (HR, 6.94; 95% CI, 3.94-12.25), primary biliary cholangitis (HR, 42.12; 95% CI, 20.76-85.44), and autoimmune hepatitis (HR, 21.26; 95% CI, 6.79-66.61) (*P* < .002 for heterogeneity). Nine immune-mediated diseases were associated with an increased risk of cancers in the involved organs (eg, asthma with lung cancer [HR, 1.34; 95% CI, 1.14-1.57; *P* < .001] and celiac disease with small intestine cancer [HR, 6.89; 95% CI, 2.18-21.75; *P* = .001]); 13 immune-mediated diseases were associated with an increased risk of cancer in the near organs (eg, Crohn disease with liver cancer: [HR, 4.01; 95% CI, 1.65-9.72; *P* = .002]) or distant organs (eg, autoimmune hepatitis with tongue cancer [HR, 27.75; 95% CI, 3.82-199.91; *P* = .001]) or in different systems (eg, idiopathic thrombocytopenic purpura with liver cancer [HR, 11.96; 95% CI, 3.82-37.42; *P* < .001]).

**Conclusions and Relevance:**

In this cohort study, immune-mediated diseases were associated with an increased risk of total cancer. Organ-specific immune-mediated diseases had stronger associations with risk of local cancers than extralocal cancers. The associations for individual immune-mediated diseases were largely organ specific but were also observed for some cancers in the near and distant organs or different systems. Our findings support the role of local and systemic immunoregulation in cancer development.

## Key Points

**Question:**

What are the profiles of cancer risk associated with immune-mediated diseases?

**Findings:**

In this cohort study of 478 753 participants, immune-mediated diseases were associated with an increased risk of total cancer. Organ-specific immune-mediated diseases had stronger associations with risk of local cancers than extralocal cancers, and many immune-mediated diseases were associated with increased risk of cancer in the involved organs and in the near and distant organs or different systems.

**Meaning:**

The findings suggest that immune-mediated diseases are associated with risk of cancer at the local and systemic levels, supporting the role of local and systemic immunoregulation in carcinogenesis.

## Teaser

This cohort study uses data from the UK Biobank to assess the associations of immune-mediated diseases with risk of total and individual cancers and of organ-specific immune-mediated diseases with risk of local and extralocal cancers.

## Introduction

Inflammation plays a pivotal role in carcinogenesis. Recent breakthroughs in cancer immunotherapy have markedly advanced our understanding about the importance of immunoregulation in cancer development. Specific mechanisms of immunoregulation in tumorigenesis have been elucidated, such as tumor-promoting inflammation, T_H_17 and regulatory T-cell (Treg)–mediated suppression of immune surveillance, inhibition of T_H_1 immunity, and local and distant tumorigenesis regulated by microbiota via alterations in the inflammatory and metabolic circuitry.^1^

Immune-mediated diseases constitute a clinically heterogeneous group of disorders, affecting up to 10% of the population worldwide.^2,3^ Several of the aforementioned immune mechanisms for cancer are also implicated in immune-mediated diseases, such as inflammation-promoting T_H_17 dominance, dysfunctional Treg surveillance, and microbiota cross talk between colonized and distant organs.^4,5^ Several immune-mediated diseases have been associated with increased risk of cancer in the involved organs, such as inflammatory bowel diseases and colorectal cancer,^6^ primary sclerosing cholangitis and hepatobiliary cancer,^7^ and celiac disease and small intestine cancer.^8^ These findings suggest a local carcinogenic effect of immune dysregulation. However, a recent study^9^ found an association of certain immune-mediated diseases with higher risk of cancer in the distant organs, such as Crohn disease with extracolonic cancer and ulcerative colitis with hepatobiliary cancer. Rheumatoid arthritis as a systemic disease has been associated with a higher risk of lymphoma and lung cancer and a lower risk of breast, colorectal, and prostate cancers.^10,11^ These findings suggest that some immune-mediated diseases may be associated with cancer risk in the distant organs or systemically beyond local organs.

To our knowledge, no studies have examined the association of organ-specific immune-mediated diseases with the risk of local and extralocal cancers. The cancer risk profiles for individual immune-mediated diseases need further characterization. Moreover, although immune-mediated diseases and cancer share some similar environmental triggers,^12^ most prior studies^7,13^ did not adjust for the lifestyle risk factors that may confound the associations. In addition, previous studies^7,13^ did not assess the less common immune-mediated diseases associated with cancer risk.

We comprehensively assessed the prospective association of 48 immune-mediated diseases with risk of total and individual cancers in the UK Biobank. We also tested the organ specificity of the associations by mapping each organ-specific immune-mediated disease with risk of local and extralocal cancers.

## Methods

### Study Participants

This cohort study was a post hoc analysis of the UK Biobank study, a prospective cohort study that aimed to investigate the genetic, lifestyle, and environmental causes of a range of diseases.^14^ Between January 1, 2006, and December 31, 2010, a total of 502 536 adults aged 37 to 73 years were recruited at 22 assessment centers throughout the UK. All participants were registered with the UK National Health Service. At the recruitment visit, participants completed a self-administered touchscreen questionnaire on sociodemographic characteristics, lifestyle exposures, medical history, and medication use and underwent physical measurements. The *International Statistical Classification of Diseases and Related Health Problems, Tenth Revision *(*ICD-10*) was used to record disease diagnoses. For the current study, we excluded participants who withdrew informed consent (n = 30) and those who had prevalent cancer at recruitment (n = 23 753). A total of 478 753 participants were included. All participants provided written informed consent. All data were deidentified. The UK Biobank received ethical approval from the UK National Health Service, National Research Ethics Service North West, the National Information Governance Board for Health and Social Care in England and Wales, and the Community Health Index Advisory Group in Scotland. In addition, an independent ethics and governance council was formed to oversee its continued adherence to the ethics and governance framework. The current study was approved by the UK Biobank. Additional ethical approval and other details are provided in the eMethods in the Supplement. This study followed the Strengthening the Reporting of Observational Studies in Epidemiology (STROBE) reporting guideline.

### Ascertainment of Immune-Mediated Diseases

We identified a total of 48 immune-mediated diseases and compared cancer risk between individuals with and without any of these diseases. For analyses of individual immune-mediated diseases, we focused on the 27 diseases with at least 100 affected individuals and at least 10 cancer cases among the affected individuals (eTable 1 in the Supplement). Among the diseases, we assessed 12 organ-specific diseases for their associations with local or extralocal cancer that was diagnosed in at least 1 participant. The diagnosis date was the date when the *ICD-10* code was first recorded in participants’ inpatient records. We required the immune-mediated disease diagnosis to be present at least 12 months before the cancer diagnosis.

### Outcome Ascertainment

The outcome of interest was the incidence of total and individual cancers. Incident cancer cases within the UK Biobank were identified by *ICD-10* codes through linkage to the national cancer registry (C01-C97). Total cancers included all cancers except nonmelanoma skin cancer (C44). For analysis of individual cancers, we restricted to those with at least 100 events (eTable 2 in the Supplement).

### Statistical Analysis

All participants were followed up from the date of recruitment until that of cancer diagnosis, death, loss to follow-up, or the end of the study period (February 28, 2019), whichever occurred first. A total of 1253 participants were lost to follow-up, and thus, 99.7% completed the study. Individuals with either prevalent immune-mediated diseases reported at baseline enrollment or incident diseases reported during follow-up were classified as the exposure group. For incident cases, patients were considered to have no immune-mediated disease until the date of the first reported diagnosis during follow-up.

We calculated hazard ratios (HRs) and 95% CIs of total and individual cancers using time-varying Cox proportional hazards regression with age as the time scale. Model 1 was adjusted for age at recruitment, sex, and ethnicity. Model 2 was further adjusted for a set of a priori determined cancer risk factors that may be associated with immune-mediated diseases, including socioeconomic status (Townsend deprivation score), educational level, total physical activity, body mass index (BMI), waist-to-hip ratio, height, smoking status and intensity, alcohol use, consumption of processed meat and oily fish, family history of cancer, and regular use of aspirin and vitamin supplements. Sensitivity analysis of medications for immune-mediated diseases and histologic finding–specific analysis are described in the eMethods in the Supplement.

For the organ-specific immune-mediated diseases, we calculated the *P* value for heterogeneity in the associations of immune-mediated diseases with local and extralocal cancers using the contrast test method based on a fully unconstrained approach developed in the competing risks framework using a cause-specific proportional hazards model.^15^ To account for multiple testing, we performed Bonferroni correction for the primary analysis on the associations of the 27 individual immune-mediated diseases with total cancer risk and considered α = .05/27 = .002 as statistically significant. We did not conduct the correction for secondary analyses on individual cancers, which were considered exploratory, and interpreted the results with caution. SAS software, version 9.4 (SAS Institute Inc) was used for all analyses. All statistical tests were 2-sided.

## Results

### Characteristics of the Study Population

A total of 478 753 participants (mean [SD] age, 56.4 [8.1] years; 54% female) were assessed in this cohort study. Most participants were White (95%), and 61 496 (13%) had at least 1 immune-mediated disease (Table 1). Compared with participants without immune-mediated diseases, participants with immune-mediated diseases were more likely to have lower socioeconomic status, higher BMI, and lower physical activity; smoke; consume processed meat and vitamins; and use aspirin. They were less likely to have a college or university education and to consume alcohol (Table 1).

**Table 1.** Age-Standardized Characteristics of Study Participants at Baseline^a^

Characteristic	Participants with immune-mediated diseases (n = 61 496)	Participants without immune-mediated diseases (n = 417 257)
Age at recruitment, mean (SD), y	57.6 (8.0)	56.2 (8.1)
Sex, %		
Female	54	54
Male	46	46
Socioeconomic status (Townsend deprivation score), mean (SD)	−0.9 (3.3)	−1.4 (3.1)
Height, mean (SD), cm	168.0 (9.4)	168.6 (9.2)
BMI, mean (SD)	28.5 (5.5)	27.3 (4.7)
Waist-to-hip ratio, mean (SD)	0.9 (0.1)	0.9 (0.1)
Total physical activity (MET), mean (SD), h/wk	42.1 (46.5)	44.6 (45.1)
Race and ethnicity, %		
Asian	3	2
Black	2	2
Mixed	1	1
White	93	94
Other^b^	1	1
College or university education, %	27	33
Smoking status, %		
Never	51	55
Previous	37	34
Current	12	10
Unknown	1	1
Alcohol consumption frequency, %		
None	12	7
Special occasions only	15	11
1-3 Times a month	12	11
1-2 Times a week	25	26
3-4 Times a week	20	24
Daily or almost daily	18	21
Unknown	0	0
Family history of cancer, %	35	35
Oily fish, occasions per week, %		
Never	13	11
<1	32	33
1	35	38
2-4	17	17
5-6	1	1
≥7	0	0
Unknown	1	1
Processed meat, occasions per week, %		
Never	9	9
<1	29	31
1	29	29
2-4	29	27
5-6	4	3
≥7	1	1
Unknown	1	0
Aspirin use, %	18	13
Vitamin supplement, %	35	31
Overall health status, %		
Excellent	7	18
Good	46	60
Fair	34	19
Poor	13	3
Unknown	0	0

Abbreviations: BMI, body mass index (calculated as weight in kilograms divided by height in meters squared); MET, metabolic equivalents.

^a^ All variables are age-standardized except age.

^b^ UK Biobank did not define “other” racial and ethnic groups.

### Any Immune-Mediated Diseases and Risk of Total Cancer

**Figure 1.**
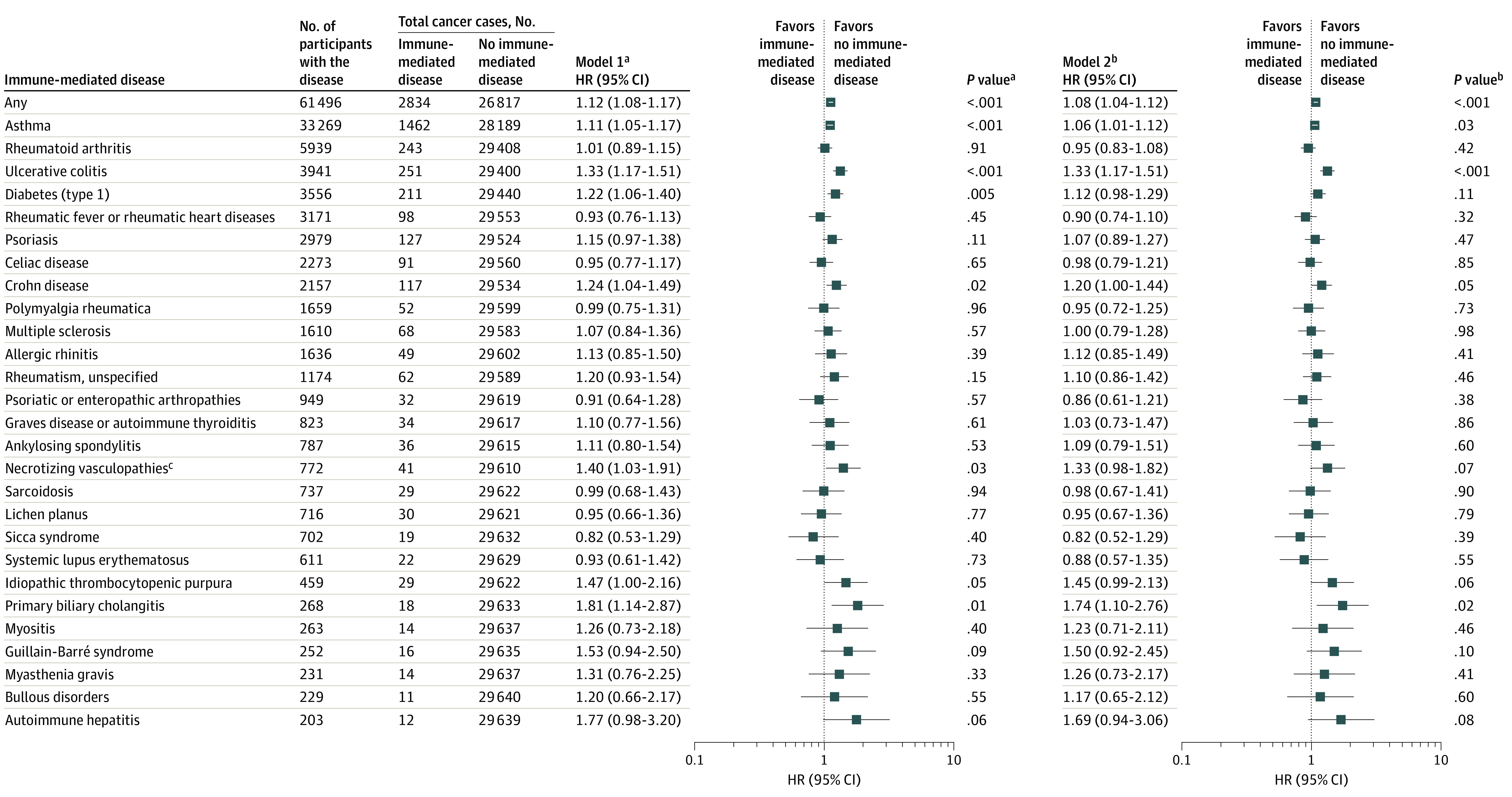
Total Cancers Associated With Immune-Mediated Diseases ^a^Model 1 was adjusted for age at recruitment, sex, and ethnicity. ^b^Model 2 was further adjusted for socioeconomic status (Townsend deprivation score), educational level, total physical activity, body mass index, waist-to-hip ratio, height, smoking status and intensity, alcohol status and consumption frequency, frequency of processed meat consumption, frequency of oily fish consumption, family history of cancer, vitamin supplements, and regular use of aspirin. ^c^Necrotizing vasculopathies except vasculitis limited to the skin.

During a total of 4 600 460 person-years and a median of 10.0 (IQR, 9.2-10.7) years of follow-up, we documented 2834 cases of cancer in participants with immune-mediated diseases and 26 817 cases in those without immune-mediated diseases. Overall, immune-mediated diseases were associated with total cancer risk after multivariable adjustment (model 2: HR, 1.08; 95% CI, 1.04-1.12) (Figure 1). Further adjustment for medication use did not substantially alter the results (HR, 1.06; 95% CI, 1.01-1.10), and self-reported medication use at recruitment was not associated with cancer risk (eTables 3 and 4 in the Supplement).

### Individual Immune-Mediated Diseases and Risk of Total Cancer

Ulcerative colitis was significantly associated with an increased risk of total cancer, with a multivariable-adjusted HR of 1.33 (95% CI, 1.17-1.51). Asthma (HR, 1.06; 95% CI, 1.01-1.12) and primary biliary cholangitis (HR, 1.74; 95% CI, 1.10-2.76) were associated with an increased risk of total cancer (Figure 1).

### Any Immune-Mediated Disease and Risk of Individual Cancers

**Figure 2.**
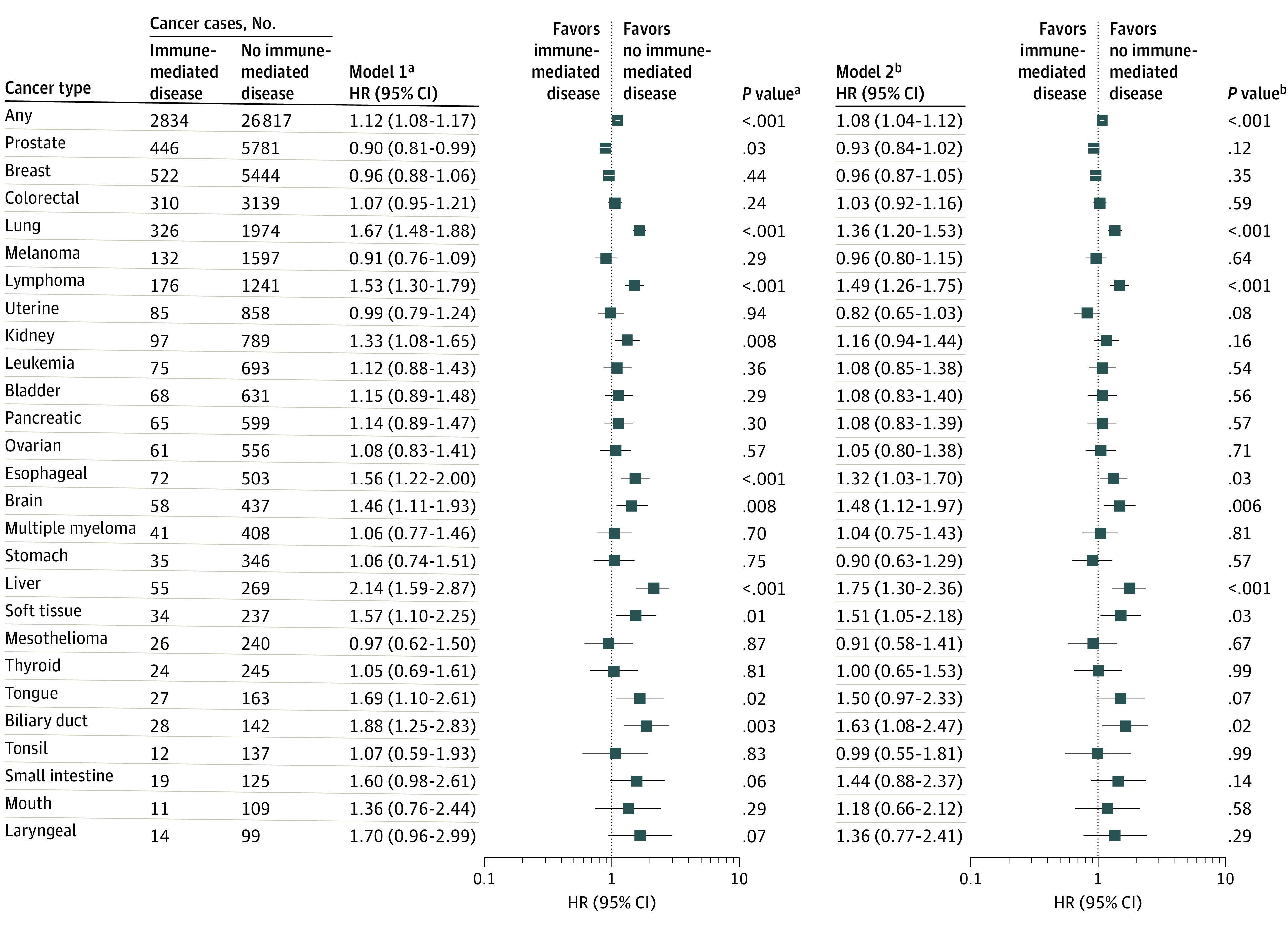
Site-Specific Cancers Associated With Immune-Mediated Diseases ^a^Model 1 was adjusted for age at recruitment, sex, and ethnicity. ^b^Model 2 was further adjusted for socioeconomic status (Townsend deprivation score), educational level, total physical activity, body mass index, waist-to-hip ratio, height, smoking status and intensity, alcohol status and consumption frequency, frequency of processed meat consumption, frequency of oily fish consumption, family history of cancer, vitamin supplements, and regular use of aspirin.

For individual cancers, participants with any immune-mediated disease were at higher risk of developing lung cancer (multivariable-adjusted HR, 1.36; 95% CI, 1.20-1.53), lymphoma (multivariable-adjusted HR, 1.49; 95% CI, 1.26-1.75), and liver cancer (HR, 1.75; 95% CI, 1.30-2.36) (Figure 2). We further assessed the association of any immune-mediated disease with risk of cancers according to histologic findings and did not find any histologic finding–specific differences (eTable 5 in the Supplement).

### Organ-Specific Immune-Mediated Diseases and Risk of Local and Extralocal Cancers

Five of the organ-specific immune-mediated diseases were significantly associated with the increased risk of local cancers, but not extralocal cancers: asthma with lower airway cancer (HR, 1.34; 95% CI, 1.14-1.56), celiac disease with small intestine cancer (HR, 6.89; 95% CI, 2.18-21.75), idiopathic thrombocytopenic purpura with hematologic cancer (HR, 6.94; 95% CI, 3.94-12.25), primary biliary cholangitis with hepatobiliary cancer (HR, 42.12; 95% CI, 20.76-85.44), and autoimmune hepatitis with hepatobiliary cancer (HR, 21.26; 95% CI, 6.79-66.61) (*P* < .001 for all comparisons; *P* < .002 for heterogeneity). In contrast, ulcerative colitis was significantly associated with higher risk of colorectal cancer and extracolorectal cancer, with a stronger association for colorectal (HR, 1.73; 95% CI, 1.26-2.39) than extracolorectal cancer (HR, 1.30; 95% CI, 1.13-1.49) (Table 2).

**Table 2.** Local and Extralocal Cancers Associated With Organ-Specific Immune-Mediated Diseases^a^

Immune-mediated disease, cancer outcome	Cancer cases, No.	Hazard ratio (95% CI)	*P* value	*P* value for heterogeneity
Asthma				
Lower airway cancer^b^	178	1.34 (1.14-1.56)	<.001	<.002
Extra–lower airway cancer	1292	1.03 (0.97-1.09)	.38	
Ulcerative colitis				
Colorectal cancer	39	1.73 (1.26-2.39)	<.001	.10
Extracolorectal cancer	217	1.30 (1.13-1.49)	<.001	
Psoriasis				
Melanoma	8	1.35 (0.67-2.71)	.40	.48
Extramelanoma	119	1.04 (0.87-1.25)	.65	
Crohn disease				
Mouth to anal cancer	27	1.34 (0.92-1.96)	.13	.46
Extra–mouth to anal cancer	91	1.14 (0.93-1.40)	.21	
Inflammatory bowel disease				
Colorectal cancer	48	1.54 (1.15-2.05)	.004	.19
Extracolorectal cancer	295	1.25 (1.11-1.40)	<.001	
Celiac disease				
Small intestine cancer	3	6.89 (2.18-21.75)	.001	<.001
Extra–small intestine cancer	89	0.96 (0.78-1.19)	.70	
Allergic rhinitis				
Upper airway cancer	1	1.08 (0.15-7.70)	.94	.97
Extra–upper airway cancer	48	1.12 (0.84-1.49)	.43	
Multiple sclerosis				
Central nervous cancer	1	0.80 (0.11-5.71)	.83	.82
Extra–central nervous cancer	67	1.01 (0.79-1.28)	.97	
Graves disease or autoimmune thyroiditis				
Thyroid cancer	1	2.76 (0.39-19.68)	.31	.32
Extrathyroid cancer	33	1.00 (0.70-1.43)	>.99	
Idiopathic thrombocytopenic purpura				
Hematologic cancer	13	6.94 (3.94-12.25)	<.001	<.001
Extrahematologic cancer	16	0.87 (0.53-1.45)	.59	
Primary biliary cholangitis				
Hepatobiliary cancer	7	42.12 (20.76-85.44)	<.001	<.001
Extrahepatobiliary cancer	11	1.07 (0.59-1.93)	.83	
Autoimmune hepatitis				
Hepatobiliary cancer	3	21.26 (6.79-66.61)	<.001	<.001
Extrahepatobiliary cancer	9	1.25 (0.63-2.50)	.53	

^a^ A Cox proportional hazards regression model was adjusted for age at recruitment, sex, ethnicity, socioeconomic status (Townsend deprivation score), educational level, total physical activity, body mass index, waist-to-hip ratio, height, smoking status and intensity, alcohol status and consumption frequency, frequency of processed meat consumption, frequency of oily fish consumption, family history of cancer, vitamin supplements, and regular use of aspirin.

^b^ Malignant neoplasm of larynx, trachea, bronchus, and lung.

### Individual Immune-Mediated Diseases and Risk of Individual Cancers

**Figure 3.**
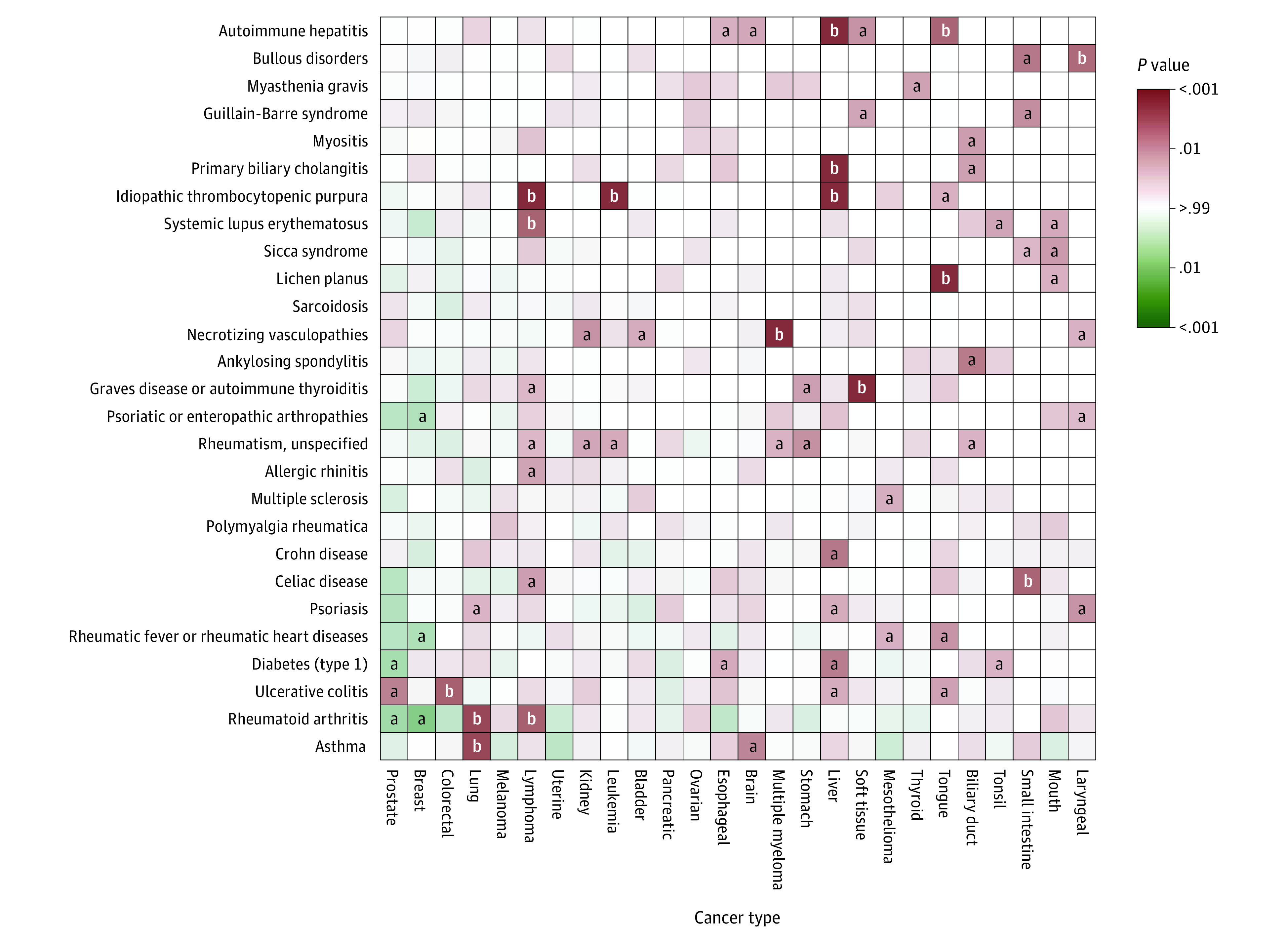
Association Profiles of Individual Immune-Mediated Diseases With Risk of Individual Cancers Red represents a hazard ratio greater than 1; green, hazard ratio less than 1. ^a^*P* = .002 to *P* < .05. ^b^*P* < .002.

Figure 3 shows the results for the site-specific analysis associating individual immune-mediated diseases with individual cancers (detailed results are presented in eTables 6 and 7 in the Supplement). Seven immune-mediated diseases were significantly associated with an increased risk of cancer in the involved organs, such as asthma with lung cancer (HR, 1.34; 95% CI, 1.14-1.57) and celiac disease with small intestine cancer (HR, 6.89; 95% CI, 2.18-21.75). The HRs ranged from 1.34 (95% CI, 1.14-1.57) to 62.42 (95% CI, 29.14-133.74) (*P* < .002). In addition, 2 associations with cancers in the involved organs were found for sicca syndrome with small intestine (HR, 8.49; 95% CI, 1.18-61.32) and mouth cancers (HR, 13.59; 95% CI, 1.86-99.09) and Guillain-Barré syndrome with soft tissue cancer (HR, 11.17; 95% CI, 1.56-79.80).

Thirteen immune-mediated diseases were associated with an increased risk of cancer in the near or distant organs or different systems. Among them, 2 were associated with increased risk of cancer in the near organs (Crohn disease with liver cancer [HR, 4.01; 95% CI, 1.65-9.72] and ulcerative colitis with liver cancer [HR, 2.59; 95% CI, 1.15-5.81]); 2 were associated with cancer in the distant organs (autoimmune hepatitis with tongue cancer [HR, 27.65; 95% CI, 3.82-199.91] and esophageal cancer [HR, 9.28; 95% CI, 1.31-65.94] and ulcerative colitis with tongue cancer [HR, 3.49; 95% CI, 1.29-9.43]). Twelve immune-mediated diseases were associated with cancers in different systems, such as idiopathic thrombocytopenic purpura with liver cancer (HR, 11.96; 95% CI, 3.82-37.42), bullous disorders with laryngeal cancer (HR, 26.23; 95% CI, 3.62-190.15), Graves disease or autoimmune thyroiditis with soft tissue cancer (HR, 9.19; 95% CI, 2.93-28.81), and ulcerative colitis with prostate cancer (HR, 1.45; 95% CI, 1.13-1.85).

Among all cancers, lymphoma demonstrated an extensive association with immune-mediated diseases. The HR ranged from 2.01 (95% CI, 1.34-3.01) for rheumatoid arthritis to 7.72 (95% CI, 3.67-16.23) for idiopathic thrombocytopenic purpura. Rheumatoid arthritis was associated with an increased risk of lung cancer (HR, 1.71; 95% CI, 1.28-2.28) and lymphoma (HR, 2.01; 95% CI, 1.34-3.01) but a decreased risk of prostate (HR, 0.62; 95% CI, 0.41-0.94) and breast (HR, 0.64; 95% CI, 0.46-0.89) cancers. Necrotizing vasculopathies as a systemic disease was significantly associated with an increased risk of multiple myeloma (HR, 7.98; 95% CI, 2.97-21.43). Type 1 diabetes was associated with an increased risk of liver (HR, 2.82; 95% CI, 1.43-5.56), esophageal (HR, 2.13; 95% CI, 1.13-4.02), and tonsil (HR, 3.57; 95% CI, 1.11-11.46) cancers but a decreased risk of prostate cancer (HR, 0.67; 95% CI, 0.46-0.97).

## Discussion

In this large prospective cohort study, we found that any immune-mediated disease was associated with a modestly increased risk of total cancer after adjusting for common cancer risk factors. Although organ-specific immune-mediated diseases had stronger positive associations with risk of local cancers than extralocal cancers, several immune-mediated diseases were associated with an increased risk of cancers in the near or distant organs or different systems. Some systemic immune-mediated diseases had a positive association with risk of some cancers but a negative association with risk of other cancers. These findings may provide insight into the local and systemic effect of immune-mediated disease in the development of cancer.

To our knowledge, the current study represents the first comprehensive effort to dissect various individual immune-mediated diseases associated with cancer risk. We found that ulcerative colitis, asthma, and primary biliary cholangitis were associated with an increased risk of total cancer. These findings are consistent with prior studies.^16,17,18^ For example, a national cohort study^16^ in Sweden found an association of asthma with a 19% increased risk of total cancer. Ulcerative colitis was associated with a 40% higher risk of total cancer, mainly colorectal and hepatobiliary cancers.^17^ Patients with primary biliary cholangitis had double the risk of total cancer and approximately 40 times higher risk of hepatobiliary cancer.^18^

Furthermore, we reported, for the first time to our knowledge, that organ-specific immune-mediated diseases were more strongly associated with higher risk of local cancers than extralocal cancers. We identified 7 immune-mediated diseases that were significantly associated with increased risk of cancer in the involved organs. These findings suggest an important role of a local carcinogenic effect of immune dysregulation. Similar associations with local cancer risk have been reported in previous studies of asthma,^19^ ulcerative colitis,^6^ primary biliary cholangitis,^7^ celiac disease,^8^ autoimmune hepatitis,^20^ idiopathic thrombocytopenic purpura,^21^ and lichen planus.^22^ In addition, we found that Sicca syndrome was associated with an increased risk of small intestine and mouth cancers and Guillain-Barré syndrome with soft tissue cancer. Regarding the mechanisms of these findings, overactivation of interleukin (IL)–12 and IL-23 signaling in immune-mediated diseases drives aberrant T_H_1 and T_H_17 immune responses, leading to chronic inflammation.^4^ The IL-23/T_H_17/IL-17 axis may reduce barrier function in the skin and local mucosa of the gut and lung and suppress cytotoxic T-cell–mediated antitumor immune surveillance in the involved organs.^23,24,25^ Immunopathology-mediated cell and organ damage, such as that induced by T_H_17 activation in inflammation, may also explain our observed associations.^26,27^

Of interest, we found that autoimmune hepatitis, Crohn disease, and ulcerative colitis were associated with increased risk of cancers in the near or distant organs within the gastrointestinal system. This finding could explain why inflammatory bowel disease and ulcerative colitis were associated with extracolorectal cancer risk in our study. Previous studies^9,17,28^ have found an association of Crohn disease and ulcerative colitis with hepatobiliary cancer. Despite the increasing evidence for the gut-liver axis^29,30^ and gut-oral axis,^31,32,33^ the observed associations of autoimmune hepatitis with tongue and esophageal cancers and ulcerative colitis with tongue cancer have not been reported elsewhere to our knowledge. We also observed an association between immune-mediated disease and risk of cancers in the different systems, such as ulcerative colitis with increased risk of prostate cancer and bullous disorders with laryngeal cancer. Consistent with our findings, 2 nationwide studies in Korea^34^ and Denmark^35^ reported an increased risk of prostate cancer in patients with ulcerative colitis but not Crohn disease. The association of aberrant fecal microbiome with ulcerative colitis^36^ and risk of prostate cancer^37^ as well as the anatomical proximity may explain the association. Although case series have reported that patients with mucous membrane pemphigoid may be susceptible to laryngeal cancer,^38^ the association of bullous disorders with laryngeal cancer risk has not been reported to our knowledge. Other positive associations of immune-mediated disease with cancers in the different systems, such as idiopathic thrombocytopenic purpura with liver cancer, Graves disease or autoimmune thyroiditis with soft tissue cancer, and psoriasis with lung and laryngeal cancer, have not been reported and need further research. The potential mechanisms include shared triggers such as hepatitis virus,^39^ herpesvirus,^40,41^ and skin, lung, and oral dysbiosis.^42,43,44^

In our study, lymphoma was among the cancers most extensively associated with immune-mediated diseases, such as idiopathic thrombocytopenic purpura, systemic lupus erythematosus, rheumatoid arthritis, and allergic rhinitis. Some of these findings have been reported in prior studies.^45,46,47,48,49^ The mechanisms may involve chronic antigenic stimulation, inflammation, and shared genetic susceptibility. Of note, compared with solid tumors, lymphoma shares more common genetic components with immune-mediated diseases.^50^

Rheumatoid arthritis, as a systemic disease, had a bidirectional association in our study, with a positive association with lung cancer and lymphoma and a negative association with prostate cancer and breast cancer. These findings are in line with previous reports.^10,11,51^ Patients with rheumatoid arthritis manifest oral and lung dysbiosis and pulmonary involvement, which could increase the risk of lung cancer.^52,53^ Previous studies^11,54,55,56^ suggest that the inverse associations with prostate and breast cancers are unlikely caused by the use of anti-inflammatory drugs, consistent with our sensitivity findings. We also found a bidirectional association of type 1 diabetes with cancer risks. Similar observations have been reported previously.^57^ The mechanisms for the bidirectional associations of type 1 diabetes and inverse associations of rheumatoid arthritis with risk of certain cancers remain unclear. Although increasing data indicate the diverse role of some immune components (eg, Tregs) and the gut microbiome in the development of different types of cancers,^58,59^ more studies are needed to characterize these immune and microbial changes in patients with immune-mediated diseases and elucidate the potential association of these changes with cancer risk.

### Strengths and Limitations

The major strengths of this study include the prospective cohort design, large sample size, and comprehensive organ-specific assessment. In addition, we adjusted for a variety of lifestyle risk factors that may have confounded the association of immune-mediated disease and cancer risk.

This study also has some limitations. First, the medications for treatment of immune-mediated diseases were self-reported by participants at recruitment only. No detailed data on dose or duration information were collected, thus precluding a detailed analysis of these medications. However, in line with our sensitivity analysis results, medications for immune-mediated diseases have not been associated with cancer risk^34,46,60,61,62^ and thus are unlikely to have had a substantial confounding effect on our results. Second, the number of cancer cases was small for some rare immune-mediated diseases. Third, despite our use of Bonferroni correction for multiple testing, chance findings could not be ruled out. Fourth, the ascertainment of immune-mediated diseases was based on inpatient records only. We may have missed some diagnoses made in the outpatient setting. However, our reported prevalence of immune-mediated disease appeared to be consistent with those in other studies in Western countries.^2,3^ In addition, given the prospective design, any misclassification in the exposure status is likely to have biased the associations toward the null. Fifth, the cohort was relatively young, with a limited number of cases of rare cancers, thus reducing the power to identify associations, particularly for metastatic cancers. Sixth, the cohort participants are predominantly White, and the findings may not be generalizable to other racial and ethnic groups.

## Conclusions

In this cohort study, overall immune-mediated diseases were associated with an increased risk of total cancer. Organ-specific immune-mediated diseases had stronger associations with risk of local cancer than extralocal cancer. Many immune-mediated diseases were associated with increased risk of cancers in the involved organs and in the near and distant organs or different systems. These findings support the importance of local and systemic immunoregulation in carcinogenesis and may inform future research elucidating the role of immunoregulation and microbiota in cancer development.

## Notes

Supplement. **eMethods.** Supplemental Methods **eTable 1.** The List of Immune-Mediated Diseases and Their ICD-10 Codes **eTable 2.** The List of Individual Cancers and Their ICD-10 Codes **eTable 3.** Self-Reported Use of Medications for Treatment of Immune-Mediated Diseases at Recruitment **eTable 4.** Hazard Ratios (95% Confidence Intervals) of Overall and Site-Specific Cancers Associated with Self-Reported Use of Anti-Inflammatory and Immunomodulatory Medications at Recruitment **eTable 5.** Hazard Ratios (95% Confidence Intervals) of Cancers Associated with Immune-Mediated Diseases According to Cancer Histology **eTable 6.** Hazard Ratios (95% Confidence Intervals) of Individual Cancers Associated with Individual Immune-Mediated Diseases **eTable 7.** Event Numbers of Individual Cancers for Individual Immune-Mediated Diseases
