# Supplementary material for: Immune-Mediated Diseases Associated With Cancer Risks
Source: JAMA Oncol. 2021 Dec 2;8(2):209–19. doi: 10.1001/jamaoncol.2021.5680 (PMC8640951; doi:10.1001/jamaoncol.2021.5680)
Supplement: Supplement. — eMethods. Supplemental Methods eTable 1. The List of Immune-Mediated Diseases and Their ICD-10 Codes eTable 2. The List of Individual Cancers and Their ICD-10 Codes eTable 3. Self-Reported Use of Medications for Treatment of Immune-Mediated Diseases at Recruitment eTable 4. Hazard Ratios (95% Confidence Intervals) of Overall and Site-Specific Cancers Associated with Self-Reported Use of Anti-Inflammatory and Immunomodulatory Medications at Recruitment eTable 5. Hazard Ratios (95% Confidence Intervals) of Cancers Associated with Immune-Mediated Diseases According to Cancer Histology eTable 6. Hazard Ratios (95% Confidence Intervals) of Individual Cancers Associated with Individual Immune-Mediated Diseases eTable 7. Event Numbers of Individual Cancers for Individual Immune-Mediated Diseases [file jamaoncol-e215680-s001.pdf]

Supplementary Online Content

He M-m, Lo C-H, Wang K, et al. Immune-mediated diseases associated with cancer risks. *JAMA Oncol.* Published online December 2, 2021. doi:10.1001/jamaoncol.2021.5680

eMethods. Supplemental Methods
eTable 1. The List of Immune-Mediated Diseases and Their ICD-10 Codes
eTable 2. The List of Individual Cancers and Their ICD-10 Codes

eTable 3. Self-Reported Use of Medications for Treatment of Immune-Mediated Diseases at Recruitment

eTable 4. Hazard Ratios (95% Confidence Intervals) of Overall and Site-Specific Cancers Associated with Self-Reported Use of Anti-Inflammatory and Immunomodulatory Medications at Recruitment

eTable 5. Hazard Ratios (95% Confidence Intervals) of Cancers Associated with Immune-Mediated Diseases According to Cancer Histology

eTable 6. Hazard Ratios (95% Confidence Intervals) of Individual Cancers Associated with Individual Immune-Mediated Diseases

eTable 7. Event Numbers of Individual Cancers for Individual Immune-Mediated Diseases

This supplementary material has been provided by the authors to give readers additional information about their work.

© 2021 He M-m et al. *JAMA Oncology.*

eMethods. Supplemental Methods

Study Participants

Ethical approval of the study

The UK Biobank received ethical approval from the NHS and National Research Ethics Service North West (11/NW/0382; 16/NW/0274). Besides, it was also approved by the National Information Governance Board for Health and Social Care in England and Wales, and the Community Health Index Advisory Group in Scotland. In addition, an independent Ethics and Governance Council was formed to oversee its continuous adherence to the Ethics and Governance Framework (http://www.ukbiobank.ac.uk/ethics/). The current study was conducted under the UK Biobank application 46466.

Informed consent of study participants

During the assessment center visit, UK Biobank staff answered questions, provided clarifications and explained the consent process. If individuals decided to take part, their signed consent was then sought and recorded before they were enrolled.

Reporting race/ethnicity

We have adjusted for various potential confounders including race/ethnicity in assessing the association of immune-mediated diseases with cancer risks. At the baseline recruitment visit, participants completed a self-administered touchscreen questionnaire on socio-demographics which contained race/ethnicity. Options for race/ethnicity were defined by UK Biobank staff.

Statistical Analysis

Missing data

Missing data on covariates were very rare (≤1% of participants for most covariates). We used missing indicators for categorical covariates and imputed missing values for continuous covariates using the median values.

Sensitivity analysis

We further described the usage of anti-inflammatory (non-steroidal anti-inflammatory drugs, NSAIDs) and immunomodulatory medications (including corticosteroids, synthetic (sDMARDs) and biological disease-modifying anti-rheumatic drugs (bDMARDs)) self-reported at recruitment in patients with immune-mediated diseases, and then we assessed associations of use with those anti-inflammatory and immunomodulatory medications at recruitment and cancer risks after multivariate adjustment.

Histology-specific analysis

Among the cancers showing an association with any immune-mediated diseases, we further assessed the associations according to cancer histological types and calculated the *P* for heterogeneity using a fully unconstrained approach developed in the competing risks framework using cause-specific proportional hazards model. [1]

eReference

1. Wang M, Spiegelman D, Kuchiba A, et al. Statistical methods for studying disease subtype heterogeneity. *Stat Med.* 2016;35(5):782-800.

© 2021 He M-m et al. *JAMA Oncology.*

eTable 1. The List of Immune-Mediated Diseases and Their ICD-10 Codes	
**Immune-mediated disease**	**ICD-10 codes**
Asthma	J45, J46
Rheumatoid arthritis	M05, M06, M08
Ulcerative colitis	K51
Diabetes mellitus (Type I)	E10
Rheumatic fever / rheumatic heart diseases	I00-I02, I05-I09
Psoriasis	L40
Celiac disease	K90.0
Crohn's disease	K50
Polymyalgia rheumatica	M35.3
Multiple sclerosis	G35
Allergic rhinitis	J301-304
Rheumatism, unspecified	M79.0
Psoriatic and enteropathic arthropathies	M07
Graves' disease / Autoimmune thyroiditis	E05.0, E06.3
Ankylosing spondylitis	M45
Necrotizing vasculopathies	M31
Sarcoidosis	D86
Lichen planus	L43
Sicca syndrome	M35.0
Systemic Lupus erythematosus	L93, M32
Idiopathic thrombocytopenic purpura	D69.3
Primary biliary cholangitis	K74.3
Myositis	M60
Guillain-Barre syndrome	G61.0
Myasthenia gravis	G70.0
Bullous disorders	L10-L14
Autoimmune hepatitis	K75.4

© 2021 He M-m et al. *JAMA Oncology.*

eTable 2. The List of Individual Cancers and Their ICD-10 Codes

Cancer	ICD-10 codes
Prostate cancer	C61
Breast cancer	C50
Colorectal cancer	C18, C19, and C20
Lung cancer	C34
Melanoma	C43
Lymphoma	C81, C82, C83, C84, C85, C86, C88
Uterine cancer	C54, C55
Kidney cancer	C64, C65
Leukemia	C91, C92, C93, C94, C95
Bladder cancer	C67
Pancreatic cancer	C25
Ovarian cancer	C56
Esophageal cancer	C15
Brain cancer	C71
Multiple myeloma	C90.0
Stomach cancer	C16
Liver cancer	C22
Soft tissue cancer	C46, C47, C48, C49
Mesothelioma	C45
Thyroid cancer	C73
Tongue cancer	C01, C02
Biliary duct cancer	C23, C24
Tonsil cancer	C09
Small intestine cancer	C17
Mouth cancer	C03, C04, C05, C06
Laryngeal cancer	C32

© 2021 He M-m et al. *JAMA Oncology.*

eTable 3. Self-Reported Use of Medications for Treatment of Immune-Mediated Diseases at Recruitment

Self-reported use of medications	Participants (%)
Anti-inflammatory medications	16325 (58)
Non-steroidal anti-inflammatory drugs (NSAIDs)	16325 (58)
Immunomodulatory medications	11596 (41)
Corticosteroids	9882 (35)
Synthetic disease-modifying anti-rheumatic drugs (sDMARDs)	2555 (9)
Biological disease-modifying anti-rheumatic drugs (bDMARDs) [a]	197 (1)

[a] bDMARDs included tumor necrosis factor (TNF) inhibitors and non-TNF inhibitors.

© 2021 He M-m et al. *JAMA Oncology.*

**eTable 4. Hazard Ratios (95% Confidence Intervals) of Overall and Site-Specific Cancers Associated with Self-Reported Use of Anti-Inflammatory and Immunomodulatory Medications at Recruitment**								
**Cancer**	**Anti-inflammatory medications [a]**				**Immunomodulatory medications [b]**			
	**Model Ⅰ [c]**		**Model Ⅱ [d]**		**Model Ⅰ [c]**		**Model Ⅱ [d]**	
	**HR (95%CI)**	***P* value**	**HR (95%CI)**	***P* value**	**HR (95%CI)**	***P* value**	**HR (95%CI)**	***P* value**
Total cancer	1.03 (0.96-1.12)	.40	0.99 (0.91-1.07)	.77	1.06 (0.98-1.15)	.17	1.07 (0.98-1.16)	.14
Prostate cancer	0.79 (0.65-0.97)	.02	0.84 (0.69-1.03)	.09	0.85 (0.68-1.06)	.16	0.83 (0.66-1.04)	.11
Breast cancer	0.94 (0.78-1.13)	.50	0.93 (0.77-1.12)	.46	1.02 (0.84-1.25)	.84	1.03 (0.84-1.25)	.79
Colorectal cancer	0.96 (0.76-1.21)	.72	0.95 (0.75-1.20)	.65	1.17 (0.91-1.50)	.22	1.16 (0.90-1.48)	.25
Lung cancer	1.43 (1.14-1.80)	.002	1.13 (0.90-1.42)	.30	1.12 (0.88-1.42)	.38	1.13 (0.89-1.44)	.31
Melanoma	0.87 (0.60-1.25)	.44	0.90 (0.62-1.31)	.59	0.94 (0.63-1.41)	.77	0.95 (0.64-1.43)	.82
Lymphoma	1.23 (0.90-1.67)	.19	1.17 (0.86-1.60)	.33	1.22 (0.88-1.69)	.23	1.23 (0.89-1.70)	.21
Uterine cancer	1.00 (0.63-1.56)	.99	0.85 (0.54-1.35)	.50	0.67 (0.39-1.13)	.13	0.66 (0.39-1.11)	.12
Kidney cancer	1.04 (0.69-1.58)	.85	0.85 (0.56-1.30)	.45	1.77 (1.16-2.69)	.008	1.83 (1.20-2.80)	.005
Leukemia	1.41 (0.88-2.28)	.15	1.35 (0.83-2.18)	.23	0.89 (0.52-1.51)	.65	0.90 (0.53-1.53)	.69
Bladder cancer	1.04 (0.63-1.72)	.88	0.97 (0.58-1.62)	.91	1.26 (0.74-2.15)	.39	1.28 (0.75-2.17)	.37
Pancreatic cancer	1.33 (0.81-2.18)	.26	1.28 (0.77-2.11)	.34	0.79 (0.45-1.40)	.42	0.79 (0.45-1.40)	.43
Ovarian cancer	0.84 (0.49-1.44)	.52	0.81 (0.47-1.41)	.46	0.88 (0.49-1.59)	.67	0.88 (0.49-1.60)	.67
Esophageal cancer	1.25 (0.77-2.02)	.37	1.16 (0.71-1.89)	.56	1.22 (0.73-2.03)	.44	1.16 (0.70-1.94)	.57
Brain cancer	0.98 (0.57-1.70)	.95	1.05 (0.60-1.83)	.86	1.09 (0.61-1.97)	.77	1.07 (0.59-1.93)	.82
Multiple myeloma	0.49 (0.24-0.98)	.04	0.46 (0.23-0.93)	.03	1.87 (0.98-3.56)	.06	1.91 (1.00-3.64)	.05
Stomach cancer	1.40 (0.70-2.82)	.34	1.16 (0.57-2.35)	.68	1.12 (0.53-2.35)	.77	1.14 (0.54-2.41)	.74
Liver cancer	1.10 (0.63-1.93)	.73	0.89 (0.50-1.57)	.68	0.97 (0.52-1.80)	.93	0.97 (0.52-1.81)	.93
Soft tissue cancer	0.99 (0.49-1.98)	.97	0.95 (0.47-1.94)	.90	1.46 (0.71-2.98)	.30	1.47 (0.72-3.02)	.29
Mesothelioma	0.65 (0.26-1.64)	.36	0.57 (0.22-1.47)	.25	1.11 (0.42-2.94)	.83	1.14 (0.43-3.02)	.80
Thyroid cancer	1.70 (0.75-3.88)	.21	1.66 (0.72-3.81)	.23	0.56 (0.20-1.55)	.27	0.56 (0.20-1.53)	.26
Tongue cancer	1.24 (0.54-2.86)	.61	1.21 (0.52-2.81)	.66	0.49 (0.16-1.46)	.20	0.46 (0.15-1.38)	.17
Biliary duct cancer	0.84 (0.38-1.89)	.68	0.73 (0.32-1.66)	.45	0.90 (0.37-2.19)	.82	0.89 (0.36-2.17)	.79
Tonsil cancer	1.17 (0.36-3.83)	.80	1.13 (0.34-3.74)	.84	0.86 (0.22-3.31)	.83	0.84 (0.22-3.27)	.81
Small intestine cancer	0.85 (0.33-2.20)	.73	0.77 (0.29-2.02)	.59	2.43 (0.95-6.25)	.07	2.42 (0.93-6.24)	.07
Mouth cancer	0.76 (0.23-2.55)	.66	0.65 (0.19-2.21)	.49	0.51 (0.11-2.38)	.39	0.51 (0.11-2.37)	.39
Laryngeal cancer	0.90 (0.30-2.70)	.85	0.74 (0.24-2.28)	.60	2.02 (0.67-6.06)	.21	1.99 (0.66-6.03)	.22

[a] Anti-inflammatory medications include non-steroidal anti-inflammatory drugs.

[b] Immunomodulatory medications include corticosteroids, synthetic disease-modifying anti-rheumatic drugs and biological disease-modifying anti-rheumatic drugs.

[c] Model Ⅰ: Adjustment for age at recruitment, sex, ethnicity, and any immune-mediated disease.

[d] Model Ⅱ: Further adjustment for socio-economic status (Townsend deprivation score), education level, total physical activity, body mass index (BMI), waist / hip ratio, height, smoking status and intensity, alcohol status and consumption frequency, frequency of processed meat consumption, frequency of oily fish consumption, family history

© 2021 He M-m et al. *JAMA Oncology.*

of cancer, vitamin supplements, and regular use of aspirin.

© 2021 He M-m et al. *JAMA Oncology.*

**eTable 5. Hazard Ratios (95% Confidence Intervals) of Cancers Associated with Immune-Mediated Diseases According to Cancer Histology [a]**					
**Cancer**	**Histological types of cancer**	**No. of cancer cases**	**HR (95%CI)**	***P* value**	***P* for heterogeneity**
Lung cancer	Small cell lung cancer	40	1.47 (1.03-2.09)	.03	.64[b]
	Non-small cell lung cancer	286	1.34 (1.18-1.52)	<.001	
	Adenocarcinoma	131	1.30 (1.08-1.57)	.006	.92[c]
	Squamous cell carcinoma	75	1.41 (1.10-1.82)	.007	
	Other types	81	1.32 (1.04-1.68)	.02	
Lymphoma	Hodgkin's lymphoma	14	1.49 (0.80-2.79)	.21	>.99
	Non-Hodgkin's lymphoma	163	1.50 (1.27-1.77)	<.001	
Esophageal cancer	Adenocarcinoma	46	1.22 (0.89-1.67)	.21	.67
	Squamous cell carcinoma	19	1.50 (0.91-2.47)	.11	
	Other types	7	1.68 (0.72-3.90)	.23	
Brain cancer	Glioma	55	1.48 (1.11-1.98)	.008	.97
	Non-glioma	3	1.45 (0.42-5.04)	.56	
Liver cancer	Hepatocellular carcinoma	27	1.82 (1.20-2.76)	.005	.20
	Intrahepatic cholangiocarcinoma	19	1.36 (0.81-2.29)	.251	
	Other types	9	3.20 (1.46-7.01)	.004	
Soft tissue cancer	Leiomyosarcoma	5	1.95 (0.73-5.18)	.18	.23
	Liposarcoma	4	3.70 (1.18-11.6)	.02	
	Other types	25	1.33 (0.87-2.02)	.18	
Biliary duct cancer	Adenocarcinoma	23	1.57 (1.00-2.48)	.05	.68
	Non-adenocarcinoma	5	2.01 (0.69-5.88)	.20	

[a] Cox regression model was adjusted for age at recruitment, sex, ethnicity, socio-economic status (Townsend deprivation score), education level, total physical activity, body mass index (BMI), waist / hip ratio, height, smoking status and intensity, alcohol status and consumption frequency, frequency of processed meat consumption, frequency of oily fish consumption, family history of cancer, vitamin supplements, and regular use of aspirin.
[b] *P* for heterogeneity for small cell lung cancer and non-small cell lung cancer.
[c] *P* for heterogeneity for small cell lung cancer, adenocarcinoma, squamous cell carcinoma, and other types.

© 2021 He M-m et al. *JAMA Oncology.*

eTable 6. Hazard Ratios (95% Confidence Intervals) of Individual Cancers Associated with Individual Immune-Mediated Diseases [a]

	Prostate cancer	Breast cancer	Colorectal cancer	Lung cancer	Melanoma	Lympho-ma	Uterine cancer	Kidney cancer	Leukemia	Bladder cancer	Pancreatic cancer	Ovarian cancer	Esophageal cancer
**Asthma**	0.93 (0.81-1.06)	1.01 (0.89-1.13)	1.05 (0.89-1.23)	1.34 (1.14-1.57)	0.85 (0.66-1.10)	1.16 (0.91-1.47)	0.76 (0.56-1.03)	1.12 (0.84-1.49)	1.01 (0.72-1.42)	0.91 (0.63-1.33)	1.14 (0.82-1.59)	1.08 (0.76-1.54)	1.32 (0.94-1.84)
**Rheumatoid arthritis**	0.62 (0.41-0.94)	0.64 (0.46-0.89)	0.68 (0.43-1.06)	1.71 (1.28-2.28)	1.40 (0.87-2.26)	2.01 (1.34-3.01)	0.55 (0.24-1.22)	1.40 (0.77-2.55)	1.07 (0.51-2.27)	1.46 (0.72-2.94)	0.64 (0.24-1.71)	1.65 (0.90-3.01)	0.18 (0.03-1.29)
**Ulcerative colitis**	1.45 (1.13-1.85)	1.10 (0.78-1.56)	1.73 (1.26-2.39)	0.86 (0.50-1.49)	0.98 (0.52-1.82)	1.46 (0.85-2.53)	1.25 (0.56-2.78)	1.70 (0.91-3.17)	0.96 (0.40-2.33)	1.46 (0.69-3.07)	0.47 (0.12-1.87)	1.52 (0.63-3.66)	1.94 (0.96-3.90)
**Diabetes (Type 1)**	0.67 (0.46-0.97)	1.22 (0.84-1.75)	1.25 (0.85-1.84)	1.33 (0.90-1.97)	0.71 (0.32-1.60)	0.98 (0.51-1.90)	1.15 (0.54-2.44)	1.33 (0.71-2.50)	1.19 (0.53-2.67)	1.60 (0.79-3.23)	0.44 (0.11-1.76)	1.14 (0.36-3.57)	2.13 (1.13-4.02)
**Rheumatic fever / rheumatic heart diseases**	0.65 (0.41-1.03)	0.44 (0.20-0.97)	1.02 (0.59-1.76)	1.39 (0.85-2.28)	0.92 (0.38-2.21)	0.73 (0.27-1.95)	1.78 (0.74-4.29)	1.32 (0.55-3.18)	1.24 (0.46-3.33)	0.63 (0.16-2.52)	0.70 (0.17-2.81)	1.77 (0.57-5.53)	0.36 (0.05-2.59)
**Psoriasis**	0.62 (0.38-1.01)	0.94 (0.58-1.52)	0.93 (0.54-1.60)	1.60 (1.04-2.47)	1.35 (0.67-2.71)	1.59 (0.82-3.06)	1.15 (0.43-3.07)	0.69 (0.22-2.13)	0.60 (0.15-2.42)	0.31 (0.04-2.18)	1.99 (0.89-4.45)	NA	1.66 (0.69-4.02)
**Celiac disease**	0.52 (0.26-1.04)	0.89 (0.57-1.40)	0.89 (0.46-1.71)	0.65 (0.27-1.57)	0.57 (0.18-1.76)	2.23 (1.19-4.15)	1.26 (0.47-3.37)	1.20 (0.38-3.72)	0.83 (0.21-3.32)	1.60 (0.51-4.98)	1.46 (0.47-4.54)	0.81 (0.20-3.26)	2.40 (0.90-6.44)
**Crohn's disease**	1.17 (0.78-1.76)	0.71 (0.41-1.22)	1.08 (0.61-1.90)	1.55 (0.96-2.51)	1.39 (0.66-2.92)	1.49 (0.71-3.13)	NA	1.66 (0.69-4.01)	0.39 (0.05-2.76)	0.41 (0.06-2.92)	1.32 (0.42-4.10)	NA	0.88 (0.22-3.52)
**Polymyalgia rheumatica**	0.89 (0.45-1.79)	0.78 (0.39-1.55)	0.93 (0.42-2.07)	1.04 (0.46-2.31)	2.15 (0.96-4.80)	1.50 (0.56-4.00)	NA	0.56 (0.08-4.00)	1.94 (0.62-6.03)	NA	2.01 (0.64-6.27)	1.55 (0.38-6.21)	0.86 (0.12-6.10)
**Multiple sclerosis**	0.54 (0.20-1.43)	1.00 (0.64-1.58)	0.83 (0.37-1.85)	0.71 (0.29-1.72)	1.64 (0.74-3.67)	1.29 (0.48-3.44)	1.33 (0.50-3.56)	1.52 (0.49-4.75)	0.63 (0.09-4.51)	2.60 (0.84-8.12)	NA	1.03 (0.26-4.14)	NA
**Allergic rhinitis**	1.06 (0.57-1.96)	0.88 (0.44-1.76)	1.56 (0.78-3.12)	0.31 (0.04-2.21)	1.11 (0.36-3.43)	2.70 (1.21-6.03)	1.99 (0.64-6.19)	2.14 (0.69-6.65)	1.68 (0.42-6.75)	NA	NA	1.07 (0.15-7.59)	1.21 (0.17-8.59)
**Rheumatism, unspecified**	0.74 (0.19-2.96)	0.77 (0.45-1.30)	0.52 (0.17-1.61)	1.20 (0.57-2.52)	0.73 (0.18-2.93)	2.38 (1.07-5.33)	0.77 (0.25-2.38)	2.94 (1.21-7.11)	3.20 (1.19-8.59)	NA	2.28 (0.73-7.11)	0.50 (0.07-3.59)	NA
**Psoriatic / enteropathic arthropathies**	0.28 (0.07-1.12)	0.14 (0.02-0.99)	1.39 (0.62-3.10)	0.93 (0.30-2.88)	0.49 (0.07-3.50)	2.23 (0.84-5.97)	1.40 (0.35-5.61)	0.77 (0.11-5.49)	NA	NA	NA	NA	1.19 (0.17-8.51)
**Graves' / autoimmune thyroiditis**	0.83 (0.21-3.31)	0.52 (0.21-1.24)	0.61 (0.15-2.45)	1.80 (0.81-4.01)	1.84 (0.59-5.71)	2.87 (1.08-7.65)	1.25 (0.31-5.00)	NA	1.41 (0.20-10.0)	1.95 (0.27-13.9)	NA	NA	NA
**Ankylosing spondylitis**	1.15 (0.64-2.08)	0.61 (0.15-2.44)	0.73 (0.24-2.27)	1.52 (0.63-3.67)	0.58 (0.08-4.13)	1.90 (0.61-5.89)	NA	NA	NA	0.98 (0.14-6.96)	NA	2.87 (0.40-20.4)	1.01 (0.14-7.20)
**Necrotizing vasculopathies**	1.64 (0.85-3.16)	0.92 (0.38-2.21)	0.85 (0.27-2.64)	0.87 (0.28-2.69)	1.29 (0.32-5.18)	0.64 (0.09-4.58)	0.88 (0.12-6.29)	3.89 (1.45-10.4)	2.36 (0.59-9.47)	3.80 (1.22-11.8)	1.25 (0.18-8.88)	NA	NA
**Sarcoidosis**	1.49 (0.74-2.98)	0.82 (0.34-1.97)	0.30 (0.04-2.08)	1.71 (0.55-5.31)	0.63 (0.09-4.47)	1.37 (0.34-5.51)	0.66 (0.09-4.67)	2.05 (0.51-8.23)	1.28 (0.18-9.09)	1.71 (0.24-12.2)	NA	NA	1.94 (0.27-13.8)
**Lichen planus**	0.58 (0.19-1.80)	1.27 (0.66-2.45)	0.54 (0.14-2.17)	1.18 (0.38-3.68)	0.57 (0.08-4.06)	1.29 (0.32-5.18)	0.80 (0.11-5.70)	0.99 (0.14-7.05)	NA	NA	2.63 (0.66-10.5)	NA	NA
**Sicca syndrome**	NA	0.82 (0.37-1.84)	0.41 (0.06-2.90)	0.90 (0.22-3.60)	0.81 (0.11-5.77)	2.71 (0.87-8.42)	0.70 (0.10-4.99)	1.65 (0.23-11.8)	NA	NA	NA	2.22 (0.55-8.89)	NA
**Systemic lupus erythematosus**	0.54 (0.08-3.86)	0.40 (0.13-1.25)	1.60 (0.60-4.28)	0.77 (0.19-3.10)	NA	4.46 (1.85-10.8)	NA	NA	NA	2.64 (0.37-18.8)	NA	NA	2.70 (0.38-19.2)
**Idiopathic thrombo-cytopenic purpura**	0.73 (0.24-2.27)	NA	1.38 (0.45-4.29)	1.94 (0.62-6.02)	NA	7.72 (3.67-16.2)	NA	NA	7.47 (2.79-20.0)	NA	NA	NA	NA
**Primary biliary cholangitis**	NA	1.74 (0.72-4.19)	NA	NA	NA	NA	NA	3.54 (0.50-25.2)	NA	NA	4.13 (0.58-29.3)	NA	5.90 (0.83-42.1)
**Myositis**	1.29 (0.42-4.00)	1.08 (0.27-4.33)	NA	1.10 (0.15-7.81)	1.75 (0.25-12.4)	3.81 (0.95-15.2)	NA	NA	NA	NA	NA	4.82 (0.68-34.3)	4.16 (0.58-29.6)
**Guillain-Barre syndrome**	1.48 (0.56-3.96)	1.82 (0.59-5.64)	1.49 (0.37-5.97)	NA	NA	NA	3.21 (0.45-22.8)	2.73 (0.38-19.4)	NA	NA	NA	5.59 (0.79-39.8)	NA
**Myasthenia gravis**	1.17 (0.38-3.63)	1.24 (0.31-4.97)	NA	1.01 (0.14-7.17)	NA	NA	NA	2.47 (0.35-17.5)	NA	NA	3.51 (0.49-25.0)	5.68 (0.80-40.5)	3.87 (0.54-27.6)
**Bullous disorders**	1.18 (0.38-3.66)	1.47 (0.37-5.89)	1.79 (0.45-7.15)	NA	NA	NA	3.72 (0.52-26.5)	NA	NA	3.48 (0.49-24.8)	NA	NA	NA
**Autoimmune hepatitis**	NA	1.08 (0.27-4.31)	NA	2.94 (0.73-11.8)	NA	3.35 (0.47-23.6)	NA	NA	NA	NA	NA	NA	9.28 (1.31-65.9)

[a] Cox regression model was adjusted for age at recruitment, sex, ethnicity, socio-economic status (Townsend deprivation score), education level, total physical activity, body mass index (BMI), waist / hip ratio, height, smoking status and intensity, alcohol status and consumption frequency, frequency of processed meat consumption, frequency of oily fish consumption, family history of cancer, vitamin supplements, and regular use of aspirin.

© 2021 He M-m et al. *JAMA Oncology.*

eTable 6. Hazard ratios (95% confidence intervals) of individual cancers associated with individual immune-mediated diseases [a] (continued)

	Brain cancer	Multiple myeloma	Stomach cancer	Liver cancer	Soft tissue cancer	Mesothelioma	Thyroid cancer	Tongue cancer	Biliary duct cancer	Tonsil cancer	Small intestine cancer	Mouth cancer	Laryngeal cancer
**Asthma**	1.68 (1.18-2.39)	0.97 (0.62-1.50)	1.09 (0.70-1.71)	1.37 (0.90-2.09)	1.14 (0.68-1.94)	0.58 (0.27-1.22)	1.24 (0.74-2.07)	1.01 (0.51-1.99)	1.44 (0.83-2.51)	0.78 (0.32-1.92)	1.67 (0.92-3.05)	0.49 (0.16-1.57)	1.27 (0.59-2.77)
**Rheumatoid arthritis**	0.82 (0.26-2.57)	1.54 (0.69-3.47)	0.29 (0.04-2.10)	1.18 (0.44-3.19)	0.82 (0.20-3.30)	0.43 (0.06-3.09)	0.42 (0.06-3.04)	NA	1.56 (0.50-4.94)	1.99 (0.49-8.13)	NA	2.91 (0.91-9.28)	2.17 (0.53-8.89)
**Ulcerative colitis**	1.27 (0.47-3.40)	1.03 (0.33-3.22)	1.15 (0.37-3.60)	2.59 (1.15-5.81)	1.85 (0.59-5.78)	1.52 (0.49-4.74)	1.33 (0.33-5.37)	3.49 (1.29-9.43)	0.83 (0.12-5.92)	2.09 (0.52-8.45)	1.05 (0.15-7.53)	1.34 (0.19-9.62)	NA
**Diabetes (Type 1)**	1.54 (0.57-4.15)	0.99 (0.31-3.09)	1.15 (0.43-3.11)	2.82 (1.43-5.56)	1.30 (0.32-5.28)	0.49 (0.07-3.55)	0.69 (0.10-4.94)	NA	2.14 (0.67-6.85)	3.57 (1.11-11.5)	NA	1.11 (0.15-8.03)	NA
**Rheumatic fever / rheumatic heart diseases**	1.77 (0.57-5.52)	1.06 (0.26-4.26)	0.53 (0.07-3.81)	1.21 (0.30-4.90)	1.11 (0.15-7.92)	3.07 (1.14-8.27)	1.33 (0.19-9.51)	4.84 (1.53-15.3)	NA	NA	NA	2.13 (0.30-15.4)	NA
**Psoriasis**	2.12 (0.79-5.69)	1.07 (0.27-4.31)	1.07 (0.27-4.30)	2.85 (1.17-6.92)	1.94 (0.48-7.84)	1.67 (0.41-6.73)	NA	NA	NA	NA	NA	1.63 (0.23-11.7)	4.86 (1.53-15.4)
**Celiac disease**	2.03 (0.65-6.33)	1.42 (0.35-5.70)	NA	NA	1.19 (0.17-8.52)	NA	NA	3.95 (0.98-16.0)	1.83 (0.26-13.1)	NA	6.89 (2.18-21.8)	3.01 (0.42-21.6)	NA
**Crohn's disease**	1.90 (0.61-5.93)	1.36 (0.34-5.46)	1.43 (0.36-5.75)	4.01 (1.65-9.72)	NA	NA	1.15 (0.16-8.22)	2.88 (0.71-11.6)	NA	1.89 (0.26-13.5)	2.05 (0.29-14.7)	2.12 (0.29-15.2)	2.16 (0.30-15.5)
**Polymyalgia rheumatica**	NA	2.11 (0.52-8.48)	NA	NA	1.98 (0.28-14.2)	NA	NA	NA	2.24 (0.31-16.1)	NA	3.44 (0.48-24.8)	5.67 (0.78-41.1)	NA
**Multiple sclerosis**	NA	NA	1.21 (0.17-8.74)	1.22 (0.17-8.74)	1.52 (0.21-10.9)	5.02 (1.24-20.3)	1.26 (0.18-9.01)	1.80 (0.25-12.9)	2.56 (0.36-18.4)	2.97 (0.41-21.4)	NA	NA	NA
**Allergic rhinitis**	2.59 (0.65-10.4)	NA	NA	NA	NA	2.71 (0.38-19.3)	NA	3.46 (0.48-24.8)	NA	NA	NA	NA	NA
**Rheumatism, unspecified**	1.36 (0.19-9.72)	3.57 (1.14-11.2)	4.91 (1.56-15.4)	NA	1.62 (0.23-11.6)	NA	2.74 (0.68-11.1)	NA	4.72 (1.15-19.3)	NA	NA	NA	NA
**Psoriatic / enteropathic arthropathies**	1.69 (0.24-12.0)	3.46 (0.86-13.9)	2.07 (0.29-14.8)	3.89 (0.96-15.7)	NA	NA	NA	NA	NA	NA	NA	6.21 (0.86-44.8)	7.76 (1.07-56.2)
**Graves' / autoimmune thyroiditis**	NA	NA	5.90 (1.47-23.8)	3.00 (0.42-21.4)	9.19 (2.93-28.8)	NA	2.76 (0.39-19.7)	5.50 (0.77-39.4)	NA	NA	NA	NA	NA
**Ankylosing spondylitis**	1.70 (0.24-12.1)	NA	NA	NA	NA	NA	4.53 (0.63-32.4)	3.59 (0.50-25.8)	8.69 (2.14-35.3)	4.89 (0.68-35.1)	NA	NA	NA
**Necrotizing vasculopathies**	2.17 (0.30-15.5)	7.98 (2.97-21.4)	NA	2.33 (0.33-16.7)	3.60 (0.50-25.8)	NA	NA	NA	NA	NA	NA	NA	9.18 (1.27-66.5)
**Sarcoidosis**	NA	NA	NA	2.58 (0.36-18.4)	3.50 (0.49-25.0)	NA	NA	NA	NA	NA	NA	NA	NA
**Lichen planus**	2.08 (0.29-14.8)	NA	NA	2.66 (0.37-19.0)	NA	NA	NA	24.3 (8.96-65.8)	NA	NA	NA	9.31 (1.29-67.0)	NA
**Sicca syndrome**	NA	NA	NA	NA	3.97 (0.56-28.4)	NA	NA	NA	NA	NA	8.49 (1.18-61.3)	13.6 (1.86-99.1)	NA
**Systemic lupus erythematosus**	NA	NA	NA	3.41 (0.48-24.4)	NA	NA	NA	NA	5.69 (0.79-40.9)	11.54 (1.60-83.4)	NA	10.9 (1.50-78.9)	NA
**Idiopathic thrombo-cytopenic purpura**	NA	NA	NA	12.0 (3.82-37.4)	NA	5.07 (0.71-36.2)	NA	9.25 (1.29-66.2)	NA	NA	NA	NA	NA
**Primary biliary cholangitis**	NA	NA	NA	62.4 (29.1-134)	NA	NA	NA	NA	12.3 (1.70-88.4)	NA	NA	NA	NA
**Myositis**	NA	NA	NA	NA	NA	NA	NA	NA	13.1 (1.82-93.9)	NA	NA	NA	NA
**Guillain-Barre syndrome**	NA	NA	NA	NA	11.2 (1.56-79.8)	NA	NA	NA	NA	NA	16.1 (2.25-116)	NA	NA
**Myasthenia gravis**	NA	5.67 (0.80-40.3)	5.03 (0.70-36.3)	NA	NA	NA	12.1 (1.69-86.6)	NA	NA	NA	NA	NA	NA
**Bullous disorders**	NA	NA	NA	NA	NA	NA	NA	NA	NA	NA	21.8 (3.04-157)	NA	26.2 (3.62-190)
**Autoimmune hepatitis**	10.8 (1.52-77.4)	NA	NA	35.1 (11.1-111)	15.1 (2.10-108)	NA	NA	27.7 (3.82-200)	NA	NA	NA	NA	NA

[a] Cox regression model was adjusted for age at recruitment, sex, ethnicity, socio-economic status (Townsend deprivation score), education level, total physical activity, body mass index (BMI), waist / hip ratio, height, smoking status and intensity, alcohol status and consumption frequency, frequency of processed meat consumption, frequency of oily fish consumption, family history of cancer, vitamin supplements, and regular use of aspirin.

© 2021 He M-m et al. *JAMA Oncology.*

eTable 7. Event numbers of individual cancers for individual immune-mediated diseases [a]

	Prostate cancer	Breast cancer	Colorectal cancer	Lung cancer	Melanoma	Lympho-ma	Uterine cancer	Kidney cancer	Leukemia	Bladder cancer	Pancreatic cancer	Ovarian cancer	Esophageal cancer
**Asthma**	224/6003	299/5667	164/3285	171/2129	61/1668	75/1342	47/896	48/838	38/730	29/670	36/628	33/584	38/537
**Rheumatoid arthritis**	20/6207	39/5927	17/3432	49/2251	16/1713	25/1392	6/937	11/875	7/761	8/691	4/660	11/606	1/574
**Ulcerative colitis**	65/6162	35/5931	39/3410	13/2287	13/1716	13/1404	6/937	11/875	5/763	8/691	1/663	5/612	8/567
**Diabetes (Type I)**	30/6197	30/5936	28/3421	27/2273	5/1724	9/1408	8/935	11/875	6/762	9/690	3/661	3/614	9/566
**Rheumatic fever / rheumatic heart diseases**	17/6210	6/5960	14/3435	15/2285	5/1724	4/1413	5/938	5/881	4/764	2/697	2/662	3/614	1/574
**Psoriasis**	17/6210	17/5949	13/3436	21/2279	8/1721	9/1408	4/939	3/883	2/766	1/698	6/658	0/617	5/570
**Celiac disease**	8/6219	20/5946	9/3440	5/2295	4/1725	10/1407	3/940	3/883	2/766	3/696	3/661	3/614	4/571
**Crohn's disease**	23/6204	14/5952	12/3437	17/2283	7/1722	7/1410	0/943	5/881	1/767	1/698	3/661	0/617	2/573
**Polymyalgia rheumatica**	8/6219	9/5957	6/3443	5/2295	5/1724	3/1414	0/943	1/885	2/766	0/699	3/661	2/615	1/574
**Multiple sclerosis**	4/6223	19/5947	6/3443	5/2295	6/1723	4/1413	4/939	3/883	1/767	3/696	0/664	2/615	0/575
**Allergic rhinitis**	10/6217	8/5958	7/3442	1/2299	3/1726	5/1412	2/941	3/883	2/766	0/699	0/664	1/616	1/574
**Rheumatism, unspecified**	2/6225	14/5952	3/3446	7/2293	2/1727	6/1411	3/940	5/881	4/764	0/699	3/661	1/616	0/575
**Psoriatic / enteropathic arthropathies**	2/6225	1/5965	6/3443	3/2297	1/1728	4/1413	2/941	1/885	0/768	0/699	0/664	0/617	1/574
**Graves' / autoimmune thyroiditis**	2/6225	6/5960	1/3448	6/2294	3/1726	4/1413	2/941	0/886	1/767	1/698	0/664	0/617	1/574
**Ankylosing spondylitis**	11/6216	1/5965	3/3446	5/2295	1/1728	3/1414	0/943	0/886	0/768	1/698	0/664	1/616	1/574
**Necrotizing vasculopathies**	8/6219	5/5961	3/3446	3/2297	2/1727	1/1416	1/942	4/882	2/766	4/695	1/663	0/617	0/575
**Sarcoidosis**	7/6220	5/5961	2/3447	3/2297	1/1728	2/1415	1/942	2/884	1/767	1/698	0/664	0/617	1/574
**Lichen planus**	3/6224	9/5957	2/3447	3/2297	1/1728	2/1415	1/942	1/885	0/768	0/699	2/662	0/617	0/575
**Sicca syndrome**	0/6227	5/5961	1/3448	2/2298	1/1728	3/1414	1/942	1/885	0/768	0/699	0/664	2/615	0/575
**Systemic lupus erythematosus**	1/6226	3/5963	4/3445	2/2298	0/1729	6/1411	0/943	0/886	0/768	1/698	0/664	0/617	1/574
**Idiopathic thrombo-cytopenic purpura**	2/6225	1/5965	3/3446	3/2297	0/1729	8/1409	0/943	0/886	4/764	0/699	0/664	0/617	0/575
**Primary biliary cholangitis**	0/6227	5/5961	0/3449	0/2300	0/1729	0/1417	0/943	1/885	0/768	0/699	1/663	0/617	1/574
**Myositis**	3/6224	2/5964	0/3449	1/2299	1/1728	1/1416	0/943	0/886	0/768	0/699	0/664	1/616	1/574
**Guillain-Barre syndrome**	4/6223	3/5963	2/3447	0/2300	0/1729	0/1417	1/942	1/885	0/768	0/699	0/664	1/616	0/575
**Myasthenia gravis**	3/6224	2/5964	0/3449	1/2299	0/1729	0/1417	0/943	1/885	0/768	1/698	1/663	1/616	1/574
**Bullous disorders**	3/6224	2/5964	2/3447	0/2300	0/1729	0/1417	1/942	0/886	0/768	0/699	0/664	0/617	0/575
**Autoimmune hepatitis**	0/6227	2/5964	0/3449	2/2298	0/1729	1/1416	0/943	0/886	0/768	1/698	0/664	0/617	1/574

[a] Each cell represents the event numbers of individual cancer in participants with and without the corresponding individual immune-mediated disease, separated by a slash.

© 2021 He M-m et al. *JAMA Oncology.*

eTable 7. Event numbers of individual cancers for individual immune-mediated diseases [a] (continued)

	Brain cancer	Multiple myeloma	Stomach cancer	Liver cancer	Soft tissue cancer	Mesothelioma	Thyroid cancer	Tongue cancer	Biliary duct cancer	Tonsil cancer	Small intestine cancer	Mouth cancer	Laryngeal cancer
Asthma	35/460	20/429	21/360	23/301	15/256	10/256	16/253	11/179	14/156	5/144	12/132	1/119	7/106
Rheumatoid arthritis	2/493	6/443	1/380	3/321	2/269	2/264	1/268	0/190	3/167	2/147	0/144	3/117	2/111
Ulcerative colitis	3/492	3/446	3/378	7/317	3/268	4/262	2/267	4/186	1/169	2/147	1/143	1/119	0/113
Diabetes (Type I)	4/491	2/447	4/377	10/314	2/269	1/265	1/268	1/189	3/167	3/146	0/144	1/119	0/113
Rheumatic fever / rheumatic heart diseases	3/492	2/447	1/380	2/322	1/270	4/262	1/268	3/187	0/170	0/149	0/144	1/119	0/113
Psoriasis	4/491	2/447	2/379	5/319	2/269	2/264	0/269	0/190	0/170	0/149	0/144	1/119	3/110
Celiac disease	3/492	2/447	0/381	0/324	0/271	0/266	0/269	2/188	1/169	0/149	3/141	1/119	0/113
Crohn's disease	3/492	2/447	2/379	5/319	0/271	0/266	1/268	2/188	0/170	1/148	1/143	1/119	1/112
Polymyalgia rheumatica	0/495	2/447	0/381	0/324	1/270	0/266	0/269	0/190	1/169	0/149	1/143	1/119	0/113
Multiple sclerosis	0/495	0/449	2/379	1/323	1/270	2/264	1/268	1/189	1/169	1/148	0/144	0/120	0/113
Allergic rhinitis	2/493	0/449	0/381	0/324	0/271	1/265	0/269	1/189	0/170	0/149	0/144	0/120	0/113
Rheumatism, unspecified	1/494	3/446	3/378	0/324	1/270	0/266	2/267	0/190	2/168	0/149	0/144	0/120	0/113
Psoriatic / enteropathic arthropathies	1/494	2/447	1/380	2/322	0/271	0/266	0/269	0/190	0/170	0/149	0/144	1/119	1/112
Graves' / autoimmune thyroiditis	0/495	0/449	2/379	1/323	3/268	0/266	1/268	1/189	0/170	0/149	0/144	0/120	0/113
Ankylosing spondylitis	1/494	0/449	0/381	0/324	0/271	0/266	1/268	1/189	2/168	1/148	0/144	0/120	0/113
Necrotizing vasculopathies	1/494	4/445	0/381	1/323	1/270	0/266	0/269	0/190	0/170	0/149	0/144	0/120	1/112
Sarcoidosis	0/495	0/449	0/381	1/323	1/270	0/266	0/269	0/190	0/170	0/149	0/144	0/120	0/113
Lichen planus	1/494	0/449	0/381	1/323	0/271	0/266	0/269	4/186	0/170	0/149	0/144	1/119	0/113
Sicca syndrome	0/495	0/449	0/381	0/324	1/270	0/266	0/269	0/190	0/170	0/149	1/143	1/119	0/113
Systemic lupus erythematosus	0/495	0/449	0/381	1/323	0/271	0/266	0/269	0/190	1/169	1/148	0/144	1/119	0/113
Idiopathic thrombo-cytopenic purpura	0/495	0/449	0/381	4/320	0/271	1/265	0/269	1/189	0/170	0/149	0/144	0/120	0/113
Primary biliary cholangitis	0/495	0/449	0/381	6/318	0/271	0/266	0/269	0/190	1/169	0/149	0/144	0/120	0/113
Myositis	1/494	0/449	0/381	0/324	0/271	0/266	0/269	0/190	1/169	0/149	0/144	0/120	0/113
Guillain-Barre syndrome	0/495	0/449	0/381	0/324	1/270	0/266	0/269	0/190	0/170	0/149	1/143	0/120	0/113
Myasthenia gravis	0/495	1/448	1/380	0/324	0/271	0/266	1/268	0/190	0/170	0/149	0/144	0/120	0/113
Bullous disorders	0/495	0/449	0/381	0/324	0/271	0/266	0/269	0/190	0/170	0/149	1/143	0/120	1/112
Autoimmune hepatitis	1/494	0/449	0/381	3/321	1/270	0/266	0/269	1/189	0/170	0/149	0/144	0/120	0/113

[a] Each cell represents the event numbers of individual cancer in participants with and without the corresponding individual immune-mediated disease, separated by a slash.

© 2021 He M-m et al. *JAMA Oncology.*
